# Automated data extraction of electronic medical records: Validity of data mining to construct research databases for eligibility in gastroenterological clinical trials

**DOI:** 10.48101/ujms.v127.8260

**Published:** 2022-01-27

**Authors:** Nora Joseph, Ida Lindblad, Sara Zaker, Sharareh Elfversson, Maria Albinzon, Øyvind Ødegård, Li Hantler, Per M. Hellström

**Affiliations:** a Department of Medical Sciences, Gastroenterology and Hepatology, Uppsala University, Uppsala; b IQVIA Sweden AB, Solna, Stockholm, Sweden

**Keywords:** Big data, data analytics, data extraction, data mining, electronic medical records

## Abstract

**Background:**

Electronic medical records (EMRs) are adopted for storing patient-related healthcare information. Using data mining techniques, it is possible to make use of and derive benefit from this massive amount of data effectively. We aimed to evaluate validity of data extracted by the Customized eXtraction Program (CXP).

**Methods:**

The CXP extracts and structures data in rapid standardised processes. The CXP was programmed to extract TNFα-native active ulcerative colitis (UC) patients from EMRs using defined International Classification of Disease-10 (ICD-10) codes. Extracted data were read in parallel with manual assessment of the EMR to compare with CXP-extracted data.

**Results:**

From the complete EMR set, 2,802 patients with code K51 (UC) were extracted. Then, CXP extracted 332 patients according to inclusion and exclusion criteria. Of these, 97.5% were correctly identified, resulting in a final set of 320 cases eligible for the study. When comparing CXP-extracted data against manually assessed EMRs, the recovery rate was 95.6–101.1% over the years with 96.1% weighted average sensitivity.

**Conclusion:**

Utilisation of the CXP software can be considered as an effective way to extract relevant EMR data without significant errors. Hence, by extracting from EMRs, CXP accurately identifies patients and has the capacity to facilitate research studies and clinical trials by finding patients with the requested code as well as funnel down itemised individuals according to specified inclusion and exclusion criteria. Beyond this, medical procedures and laboratory data can rapidly be retrieved from the EMRs to create tailored databases of extracted material for immediate use in clinical trials.

## Introduction

Implementation of electronic medical records (EMRs) in the healthcare system has the primary ambition to facilitate registration and sharing of patient data between different healthcare units and maintain backup information for health providers (1–3). With the growth and global adoption of EMR systems in medical healthcare, massive amounts of information have become available, motivating exploration of alternative methods to make full use of these large datasets (4).

Methodologies to retrieve and analyse huge datasets, commonly described as Big Data, already exist and have evolved for over a decade in order to make data more accessible for, among others, statistical description and evaluation. Data mining techniques are being evaluated in epidemiologic studies to create local and national registers for surveillance of disease progression, for drug prescriptions, to obtain a clear view of medical healthcare in practice or to evaluate how diagnostic and treatment guidelines are applied in clinical practice (5–10). These methodologies have hitherto not been integrated as a part of research to find patients eligible for studies (10–12).

To enable efficient extraction of data, it is necessary that automated processes are validated. Prerequisites for such automation seem already to be in place using data extraction models (13–14), one of which is the Customized eXtraction Program (CXP; IQVIA Stockholm, Sweden) as used in the present study. A standardised way to access medical record units, requires an established format where all available patient information should be synoptically summarised in a uniform manner (15), providing the basis for automated EMR extractions. Adoption of data mining in research studies has previously only been possible to achieve by integrated mining software in order to search for structured data which is stored in fixed-mode databases and contains basic subject information, whereas unstructured data has to be searched for and be manually assessed (16) (see Table 1).

**Table 1:** The index terms in the form of structured and unstructured data that the CXP software is capable of extracting from electronic medical records of patients.

Structured data	Unstructured data
• Demographics:	• Imaging:
Gender	Radiology
Birth data	Computerised tomography
	Magnetic resonance imaging
	Scintigraphy
• Diagnosis:	• Laboratory data:
Primary	Microbiology
Secondary	Pathology
• Measurements:	• Case notes
Blood pressure	Text body of electronic medical records
Heart rate	
Breathing rate	
Body weight and height	
• Medication:	• Referrals:
Prescribed	Between health care providers
Administered	
• Procedures:	
Surgical	
Medical	
• Laboratory data:	
Clinical chemistry and pharmacology	

The CXP is a data mining software that can process, identify and extract structured and unstructured data inside EMRs in one single standardised process (16, 17). The CXP can be tailored to perform complex tasks of extracting information and organising specific data from different data sources such as the EMR used in primary care and hospital-based secondary healthcare environments, as well as for medical investigations and therapeutic measures (7, 16). By exploiting data mining techniques, it becomes possible to make full use and derive benefit from the significant amount of data that is available from EMRs (8, 9, 16).

The aim of our study was to investigate sensitivity of the CXP software for potential adoption into data mining techniques on the local EMR system (Cambio Healthcare Systems AB, Stockholm, Sweden) in Region Uppsala with the purpose of creating a database of relevant participants for clinical research studies of ulcerative colitis (UC) in clinically active phase. We used the International Classification of Disease-10 (ICD-10) diagnosis K51 for UC as the basis for a patient search, superimposed by inclusion and exclusion criteria in order to extract relevant subjects for the clinical study and to evaluate the validity of the CXP for finding patients eligible for inclusion in our clinical study by using structured EMR data.

## Materials and methods

### Data extraction

The CXP software (version 4.69) contains three components: Core, Adapter and CXP-online. The Core contains the logic that provides the base functionality. The Adapter is a system component that can be adapted and customised for use with different EMR source systems. In our study, the Adapter was customised for reading of the Cambio Cosmic (Cambio Healthcare Systems AB) EMR system. The CXP-online component enables configuration of the extraction as well as running the operative process and monitoring of the EMR extractions remotely.

Prior to the CXP extraction, the target diagnosis over a specific period was formalised from 2005 to 2019. From that subset of data, the target objects were then defined with index terms in the CXP. The index terms are defined variables for the inclusion and exclusion criteria, based on the Anatomic Therapeutic Chemical (ATC) coding, ICD coding and specific laboratory values according to their reference limits. By using these criteria, CXP extracted the applied index terms as listed in Table 1. The unstructured data was not manually assessed in this study.

### Data extraction and cleaning

**Figure 1:**
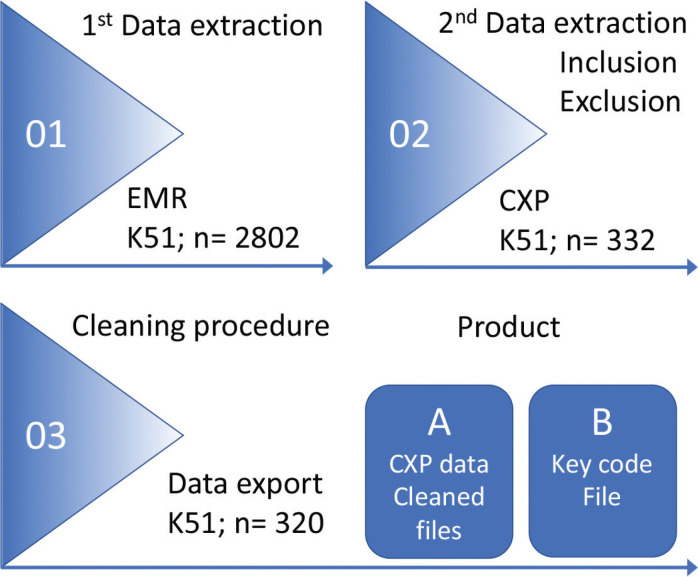
The CXP computerised process from extraction to end-product. *Note*: 1) Initial extraction, the search for K51 (UC) in all EMRs in Uppsala; (n = 2,802). 2) Extraction of target objects according to the inclusion and exclusion criteria (n = 332). 3) Cleaning procedure when all raw data is pseudonymised. Then, the pseudonymised data are exported to form a clean secure a database (A, CXP data) and a Key code file (B) containing personal identifiers and the link between the pseudonymised data and the individual EMR (stored separately).

After data extraction, according to the specific diagnosis (step 01), the first study specific extraction was done according to the inclusion and exclusion criteria (step 02), followed by the next step where the CXP removed all personal identifiers (step 03) (see Figure 1). Extracted data from the EMR meeting all index terms, was stored in a clean CXP data file and a parallel Key code file. The CXP data file contains pseudonymised personal data without any personal identifiers. All patients are coded with an internal study patient identification number. The key code file contains the link between each personal identity number/social security number and the internal study patient identification number.

### CXP data files

On the basis of the clinical trial GA29103, GARDENIA, ‘A study comparing the efficacy and safety of etrolizumab to infliximab in participants with moderate to severe UC who are naïve to tumour necrosis factor (TNF) inhibitors’ (initiated and sponsored by Hoffman-La Roche, Basel, Switzerland), we conducted a data mining project using the EMR of Region Uppsala for identifying eligible patients diagnosed with UC for the study. The clinical trial was carried out worldwide between December 2014 and June 2020.

The target population was anti-TNFα-naïve patients with moderately-severe active UC, excluding ulcerative proctitis; ICD code K51, excluding ICD-10-clinical modification code K51.2. The subjective criteria for the disease activity was based on the partial Mayo Clinic score of UC (18) with findings of blood in the stools (Mayo subscore ≥ 1), along with 3–4 stools more than normal per day (Mayo subscore ≥ 2) as revealed in the unstructured case notes of the EMR. Several inclusion and exclusion criteria, listed in Table 2, where applied as index terms in the CXP to retrieve the target population for the study.

**Table 2:** Inclusion and exclusion criteria forming the target population for the research study Ga29103, GARDENIA.

Inclusion criteria	Exclusion criteria
Moderately to severely active UC as determined by the Mayo Clinic Score	A history of current conditions and diseases affecting the digestive tract, including UC, indeterminate colitis, suspicion of ischemic, radiation or microscopic colitis, Crohn’s disease, fistulas or abdominal abscesses, colonic mucosal dysplasia, intestinal obstruction, toxic megacolon or unremoved adenomatous polyps
Gender: men and women	Prior or planned surgery for UC
Age: 18–80 years	Past or present ileostomy or colostomy
Naïve to treatment with any TNF inhibitor therapy (including TNF inhibitor biosimilars)	Have received non-permitted inflammatory bowel disease (IBD) therapies (including infliximab, adalimumab, golimumab, ustekinumab, certolizumab, natalizumab, vedolizumab, eflizumab, or tofacitinib)
Inadequate response to or intolerance of prior corticosteroid and/or immunosuppressant treatment	Chronic hepatitis B or C infection, human immunodeficiency virus (HIV) or tuberculosis (active or latent)
Background regimen for UC may include oral 5-aminosalcylate, oral corticosteroids, budesonide multi-matrix system, probiotics, azathioprine, 6-mercaptopurine, or methotrexate if doses have been stable during the screening period	History of moderate or severe allergic anaphylactic/anaphylactoid reactions to chimeric, human, or humanised antibodies; fusion proteins, or murine proteins; hypersensitivity to etrolizumab or any of its excipients
Use of hormonal contraception during and at least 24 weeks after the last dose of the study drug	

*Note*: Patients fulfilling the inclusion but not the exclusion criteria were extracted from the EMR using the structured data in CXP.

### Calculations and statistics

In order to evaluate the validity of the data that was extracted and filtered by the CXP, the retrieved data files were manually controlled against the original EMRs. The extracted data included target objects meeting all index term (*n* = 332), thus meeting all inclusion- but no exclusion criteria. Separately, all structured data extracted from each individual EMR, such as diagnosis, supportive procedures (sigmoidoscopy, colonoscopy and patient consultations), and laboratory values were used to create individual CXP files. However, these separate files are still connected to their specific target object through the internal study patient identification number.

Examination of the separate structured data was restricted to diagnosis (*n* = 4,077) and procedures (*n* = 2,364). The sensitivity of the CXP extracted structured data was evaluated, but also used as validity parameters in the study for the extracted target objects (*n* = 332). In order to carry out this examination all data was sorted according to the CXP dataset for internal patient identification. This was done manually in a separate MS Excel spreadsheet (Microsoft, Redmond, WA, USA). The EMR belonging to the internal patient identification dataset of the CXP was retrieved using the key code file. The recovery rate of eligible patients by the CXP extraction as well as the sensitivity between CXP-extracted data and manually assessed data in the original EMR source were calculated using basic sensitivity analysis.

### Ethical considerations

The study was covered by ethics approval of the Regional Ethics Review Authority, Uppsala to the study GA29103, GARDENIA; EudraCT number 2013-004282-14.

## Results

**Figure 2:**
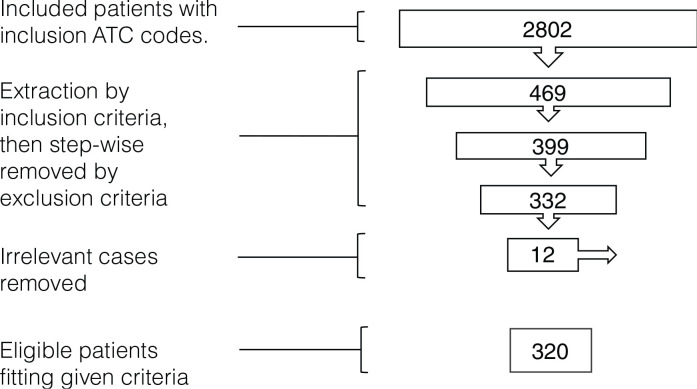
Inclusion and extraction criteria applied under the CXP. *Note*: CXP identifies patients that meet the inclusion criteria, diagnosis with ICD-code K51 (n = 2,802), in the initial extraction. A step-by-step exclusion removal process is then applied in order to funnel down and remove individuals with different exclusions (n = 469, 399) to provide a clean base of eligible patients (n = 332). In the manual examination 12 extracted target objects that meet the exclusion criteria. This resulted in a final outcome of 320 patients fitting a true eligibility according to the study criteria.

In the initial extraction step, the CXP filtering process gave 2,802 UC patients diagnosed with moderately-severe UC (all K51, excluding K51.2). The following cleaning process that was pursued in a step-by-step exclusion process revealed a total of 332 cases that fulfilled the inclusion, but not the exclusion criteria (Figure 2). In the manual reading of the EMRs of the 332 extracted cases revealed 12 individuals who were identified as irrelevant for the search. Four of these had Crohn´s disease and six had prior surgery for UC; another two cases had previously received treatment with TNFα-inhibitors as an exclusion criterion. With this approach, the number of patients who were not identified among the 332 extracted cases is not known. Hence, the CXP software extracted a total of 332 cases that was further funnelled down to 320 relevant cases after detailed ocular reading of each patient’s EMRs.

In the separate CXP-extracted structured data files, a total of 4,077 diagnoses were extracted with the CXP software from the EMRs. From the original EMR source, 4,182 diagnoses were identified and compared to the date of the CXP extracted diagnosis. Manual scrutinisation of the CXP extracted data revealed 25 duplicated diagnoses and 21 non-existent diagnoses in the original EMR source. In addition, 151 diagnoses were found in the EMR but not in the CXP data files; these were identified as missed diagnosis. In conclusion, arriving at a number of 4,031 remaining and correctly extracted diagnoses, data showed a sensitivity of 96.4% for diagnosis, with a recovery of 97.5% and over-recovery (duplicates and non-existent in original EMR source) of 1.1% (see Figure 3A). Furthermore, 2,364 procedures were examined in the separate CXP-extracted data files. Analysis revealed nine of which were duplicated and six non-existent in the original EMR source, presenting an over-recovery of 0.6%. In the original EMR sources 2,458 procedures were found, however the CXP missed 109 procedures in the extraction process, resulting in 2,349 correctly extracted and remaining procedures. The sensitivity was calculated to 95.6% for procedures (see Figure 3B). Taken together, the conjoint weighted average for sensitivity of diagnosis and procedures was estimated to 96.1%.

**Figure 3A:**
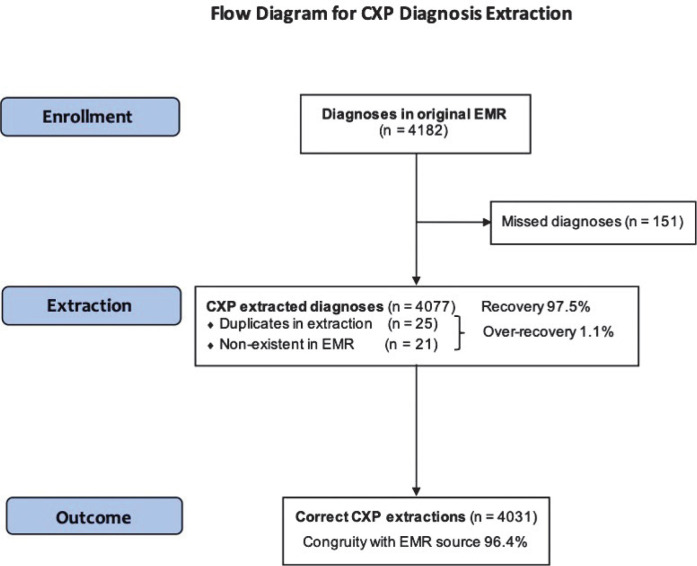
Flow diagram for the CXP procedure extraction with all diagnoses found in the EMR. *Note*: CXP extracted structured data with all diagnoses found in the EMR of the target population. The numbers of missed, duplicated and non-existing diagnoses have been removed step-by-step to arrive at a final number of correct extractions as compared with the EMR.

**Figure 3B:**
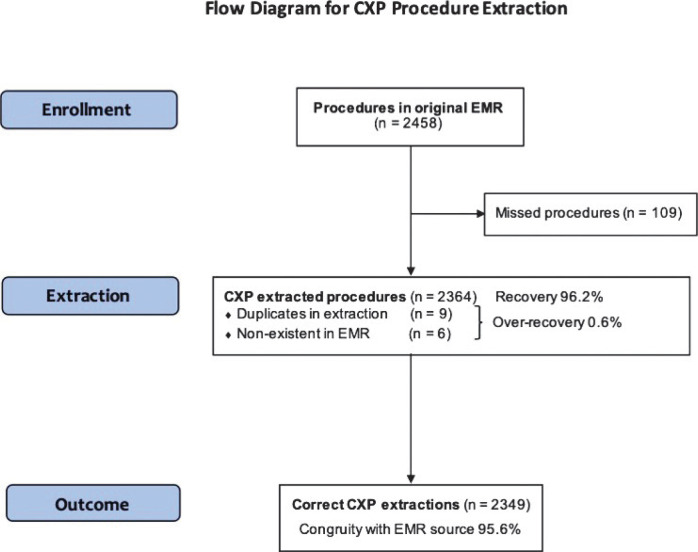
Flow diagram for the CXP procedure extraction of coherent medical procedures. *Note*: Extracted CXP data of coherent medical care procedures. The numbers of missed, duplicated and non-existing procedures have been removed step-by-step to arrive at a final number of correct extractions as compared with the EMR.

## Discussion

This novel data mining method proved feasible, enabling large rapid data extraction, with sufficiently high reliability and accuracy in order to facilitate academic studies and clinical trials (1, 2). We achieved 96.1% sensitivity with a recovery rate in the range from 95.6 to 101.1% for extracted structured data. Thus, the agreement of data is within reasonable boundaries to permit the use of the CXP software for data extraction purposes as estimated for all sorts of structured data. However, narrative unstructured data in the free text body of the EMR was not evaluated in this study since that type of data contains many soft variables that can be interpreted differently and do not permit any verifiable truth. This means that the EMRs contain personal information of various kind that will influence the eligibility of the patient for participation in a study. This type of information has to be read and interpreted for each individual in order to consider the patient’s actual ability to participate in a clinical study.

Automated extraction from different EMR sources are today used for surveillance and are utilised to create modern epidemiological datasets and for hypothesis-generating studies of disease associations and comorbidity patterns. In similar fashion, structured, and unstructured data from the past and present has the potential to be mined and analysed in order to find relevant objects for scientific studies. Modern computerised methods enable data mining of EMRs to facilitate identification of suitable patients for various studies and clinical trials. Conventional manual methods of scrutiny of large complex datasets by the naked eye are overly time-consuming and untimely to pursue (4, 5).

An advantage of the CXP software is its ability to combine different data sources and extract relevant data in one single automated extraction. With our presented data of a high and reliable recovery, the need for manual data extraction decreased significantly, as the CXP extraction is a time-saving and effective tool to promptly obtain and sort datasets from EMR sources. However, data is difficult to retrieve and directly analyse as the EMR is constructed from different software platforms, having great diversity, redundancy, incompleteness and is regulated by judicial patient data protection security as presented in the General Data Protection Regulation (GDPR). Attention should always be given to the requirement of anonymity and privacy protection for the patient (3). The pseudonymisation executed by the CXP data mining software, in which personally identifiable information fields within a data record are replaced by artificial identifiers, or pseudonyms, and with the key code file only accessible for the principal investigator should meet this demand without compromising the EMR data.

When performing data extraction, there are certain issues with the inclusion/exclusion criteria processing. For example, when tailoring the algorithm to include patients with UC, but exclude patients with Crohn’s disease, the CXP mining software searches all available data for a set diagnosis of Crohn’s disease and automatically excludes EMRs with such documented diagnosis. Due to the fact that patients often initially receive a different ‘work-up’ diagnosis for hypothesis testing before the final diagnosis is made, loss of target objects will appear leading to an underestimation of true numbers. A diagnosis could also change over time as more information is obtained and symptoms develop differently than at an early stage of the disease.

In our set of extracted data, the CXP found a higher number of diagnoses, identified as duplicates and non-existent, as compared to the scrutinised EMRs. The existing version of the software does not recognise if data is manually deleted from the EMR source by its users due to restrictions in the Cambio Intelligence database, applied by the system owner. This is a likely explanation for the discordant findings of diagnosis and procedures identified with the manual data scrutiny of the EMR. The impact of such errors should be considered when data extracted with the CXP is used for drawing final conclusions. In order to safeguard a maximally retrieved database, data must link to not only the local EMR, but also the patient register of the Swedish National Board of Health and Welfare, as applicable in Sweden, but may be linked to corresponding national registers in other countries in order to retrieve a full data set.

As mentioned in the introduction, automated data extraction requires information in the EMR to be documented in a uniform manner among all caregivers. Inconsistency negatively affects the final results of the data extraction and is potentially a loss of useful and relevant data. Similar observations were presented by Martinell and co-workers (16).

As discussed previously, the CXP software was not evaluated in this study with regard to the complex extraction of narrative unstructured data in case notes. For example, when the study population is identified as patients with ‘ulcerative colitis’ and the term ‘bloody diarrhoea’ is found in the unstructured case notes, the CXP is designed to search and recover that term as a way to consolidate the diagnosis. A potential error source of such a requested term is the negation of its presence, such as ‘patient has not had bloody diarrhoea’. Hence, the term that the CXP software is searching for exists, resulting in an unwanted data extraction. The complexity of the unstructured EMR data is a hurdle for the software to the extraction process that may require additional programming in order to retrieve accurate data. However, this approach should also be of value if patients with active disease are in focus of the research project (4).

The use of a data-driven approach by CXP enables rapid establishment of a dataset for patient recruitment, which can be optimised for different types of clinical studies according to their respective inclusion and exclusion criteria (10). Our experience is that with a conventional recruitment approach at patient encounters, or recruitment among colleagues, results are meagre. By the use of CXP, we could identify about 300 potential recruits. The study has given the staff a good insight into an effective recruitment process for clinical studies once the patient records and clinical notes are carefully written with a specific diagnosis according to known physical or laboratory biomarkers of the disease.

It can be concluded that the CXP software can be considered as a useful tool in clinical research being highly efficient in extracting relevant target objects for research. The quality of the extracted data for diagnosis and procedures showed a convincing sensitivity for structured data. Thus, the use of CXP automated extraction software is a reliable and efficient way to identify and create databases with eligible cases suitable for research studies and clinical trials.
